# Cone Beam Computed Tomography Evaluation and Proposal of a Functional Radiographic Classification of the Coronoid Process—A Cross-Sectional Study

**DOI:** 10.3390/jcm14051623

**Published:** 2025-02-27

**Authors:** Archna Nagpal, Aditya Tadinada

**Affiliations:** Division of Oral and Maxillofacial Radiology, School of Dental Medicine, University of Connecticut, 263 Farmington Avenue, Farmington, CT 06030, USA; tadinada@uchc.edu

**Keywords:** classification, cone beam computed tomography (CBCT), coronoid process, functional, hyperplasia, radiographic, zygoma

## Abstract

**Background:** The objective of this study is to investigate the cone beam computed tomography (CBCT) features of the coronoid process in patients without limitations in mouth opening and to develop a functional classification of the coronoid process, considering its relationship with the zygomatic bone. **Methods:** This cross-sectional retrospective study analyzed the CBCT features of 408 coronoid processes. Volume rendered and axial images were evaluated to assess the shape, surface configuration, length of the coronoid process, the coronoid/condyle ratio, the distance of the coronoid process from the posteromedial surface of the zygoma, and its vertical level. The coronoid process was considered hyperplastic when the coronoid/condyle ratio was >1. **Results:** The maximum coronoid processes had a triangular shape (221). The mean length of the coronoid process was 13.85 mm. The mean coronoid/condyle ratio was 0.84. A total of 85 coronoid processes were hyperplastic. The mean distance from the coronoid process to the posteromedial surface of the zygoma was 15.99 mm, ranging from 5.8–27.9 mm. The mean vertical level of the coronoid process in the study sample was 9.6 mm. A novel functional radiographic classification was developed. The coronoid processes were classified as Type I, II, III (a,b,c), and IV (a,b,c). Type IIIa was the most common (45.83%), followed by Type II (29.68%), Type I (16.17%), Type IIIc (4.41%), Type IIIb (3.18%), Type IVa (0.49%), and Type IV b (0.24%). **Conclusions:** The vertical extension of the coronoid process beyond the lower border of the zygomatic bone/arch and its distance from the posterior surface of the body of the zygoma could play a significant role in impacting the mandibular movements.

## 1. Introduction

The coronoid process is a beaklike process in the superior-anterior portion of the mandibular ramus [1]. A wide range of anatomical and morphological alterations have been reported regarding the shape, length, and width of the coronoid processes [2]. Studies undertaken by Isaac, Narayana, and P. Ranganath have observed specific forms of coronoid processes in human mandibles, e.g., triangular, rounded, hook shaped, rectangular, and flattened [3]. One of the morphological variations includes coronoid process hyperplasia, which is an abnormal elongation of the coronoid process because of formation of histologically normal bone [4]. There is restricted mouth opening, with no clinical symptoms in coronoid hyperplasia. The various proposed etiologies include developmental changes, bone pathologies, increased temporalis muscle activity, genetic abnormalities, hormonal changes, and trauma [5].

The condition of the elongated coronoid process is often overlooked, and these patients are often incorrectly treated for temporomandibular joint (TMJ) disorder, since the entire focus is on the mandibular condyle and the glenoid fossa relationship of the TMJ [6]. The large coronoid process can interfere with the medial or temporal surface of the zygomatic bone during mandibular movements. The infratemporal space required for the rotation and translation of the coronoid process of the mandible is reduced, which leads to limitation in the range of mandibular movement during mouth opening and lateral excursions [1].

The differential diagnosis of coronoid hyperplasia includes unilateral coronoid osteomas and osteochondromas [4] and at times, ankylosis and pseudo ankylosis of the temporomandibular joint [7]. One of the main concerns regarding the elongated coronoid process is Jacob’s disease, in which a new synovial joint is formed between the hyperplastic coronoid process and the inner aspect of the zygomatic arch [8]. The mandibular coronoid process may also fuse with adjacent bones like the maxillary, zygomatic, or temporal bones, resulting in extra-articular ankylosis [9]. Zygomatico–coronoid ankylosis is a rare clinical condition, with a bony or fibrous ankylosis between the coronoid process and the zygomatic arch [10].

Clinically, not all the coronoid processes with abnormal proliferative elongation will manifest as limited mouth opening. The reason behind this is that the temporalis muscle is not primarily affected, or there is no impingement between the elongated coronoid process and the ipsilateral zygomatic arch during mandibular movements [11]. In 2014, Ilguy concluded that instead of the length, the distance between the coronoid process and the inner face of the frontal part of the zygomatic bone may be the actual reason behind limited mouth opening [12]. The cause of restricted mandibular movement is associated with the impingement of the elongated coronoid process to the posterior aspect of the zygomatic bone or to the inside of the zygomatic arch [13].

Paucity and gaps in the literature regarding the imaging features of coronoid processes are evident. Only a few studies, such as those by Tavassol, F. et al. [6], Izumi, M. et al. [13], Isberg, A. et al. [14], and Erdem, S. et al. [15], have assessed the radiographic features of the mandibular coronoid processes. To the best of our knowledge, no study has been conducted evaluating the distance of the coronoid process to the body of the zygoma. One way to do this is to evaluate the relationship of the coronoid process to the adjacent structures in subjects with no restrictions in mouth opening.

It is important for oral and maxillofacial radiologists (OMFRs) to be watchful regarding the relationship between the zygomatic bone and coronoid process while reviewing cone beam computed tomography (CBCT) scans and to advocate appropriate referrals, if needed. Currently, there is no universally accepted classification system for the mandibular coronoid process focusing on the relationship of the coronoid process with the adjacent zygomatic bone. Hence, this study was conducted with the purpose of evaluating the morphologic form and length of the coronoid process, the coronoid/condyle ratio, the vertical level, and the distance of the coronoid process from the posteromedial surface of the zygoma on CBCT scans in patients with no limitations in mouth opening. We propose a functional classification of coronoid processes, considering the relationship of the coronoid process with zygomatic bone on a CBCT scan.

## 2. Materials and Methods

This retrospective and observational study was granted a waiver from the Institutional Review Board of UConn Health, Connecticut, USA (PG1520, 12/21/2023). A total of 204 anonymized CBCT scans of patients with no restrictions in mouth opening were obtained from the archives of UConn School of Dental Medicine in a random manner. The CBCT scans were selected considering the following criteria: CBCTs that were acquired in a closed mouth position with a field of view (FOV) of 140 × 100 mm and 170 × 120 mm, capturing the coronoid processes and the TMJs. The scans included were acquired for treatment of varying dental conditions. The CBCT scans that were acquired with the teeth in intercuspation were included in the study. The CBCT scans with radiographic evidence of TMJ abnormalities, completely edentulous arches, orofacial trauma or syndromes, and any artifacts obscuring the region of interest were excluded from the study. A total of 500 large field of view CBCT volumes were assessed, and based on the selection criteria, 204 CBCT volumes were included in the study. The exclusion rate was 59.2%.

CBCT scans were acquired by a 3D Accuitomo (J Morita Corp., Kyoto, Japan) device, with acquisition parameters of 90 kVp, 7 mA, and 17.5 s and a voxel size of 250 µm. The CBCT volumes were analyzed using Xelis Dental-3D Viewer dental imaging software (2.0.3.0 BN 3). The CBCT features were assessed by two radiologists. They were calibrated by a board certified oral and maxillofacial radiologist, with 15 years of experience in the analysis of the features of the coronoid processes. A consensus meeting was held, and both the observers reached an agreement regarding the measurements involved. Inter- and intra-observer variability was assessed to evaluate the reproducibility and reliability of the measurements.

**Figure 1:**
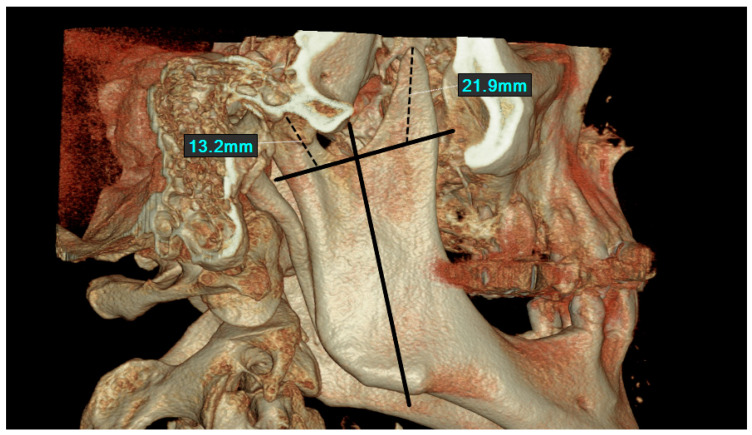
Evaluation of shape, surface configuration, and length of coronoid and condylar process on volume rendered image.

Volume rendered and axial reconstruction images were used to perform the measurements. The long axis of the ramus of the mandible was traced. A line was drawn perpendicular to the long axis and tangential to the deepest point of the sigmoid notch. The long axes of the coronoid and condylar processes were formed (Figure 1).

CBCT images were evaluated for the following features:

1. Shape of the coronoid process. This was categorized as triangular, rounded, hooked, or flattened. If the shape did not fall into any of the said categories, it was designated as miscellaneous.
2. Surface configuration of coronoid process. This was considered smooth or irregular.
3. Length of the coronoid process (Cor-L). This was evaluated along the long axis of the coronoid process. The distance between the tangent to the sigmoid notch and the tip of the coronoid process was measured.
4. Length of the condylar process (Con-L). This was analyzed along the long axis of the condylar process. The distance between the tangent and the sigmoid notch and the tip of the condylar process was measured.

The features from 1–4 were evaluated on volume rendered CBCT images (Figure 1).

**Figure 2:**
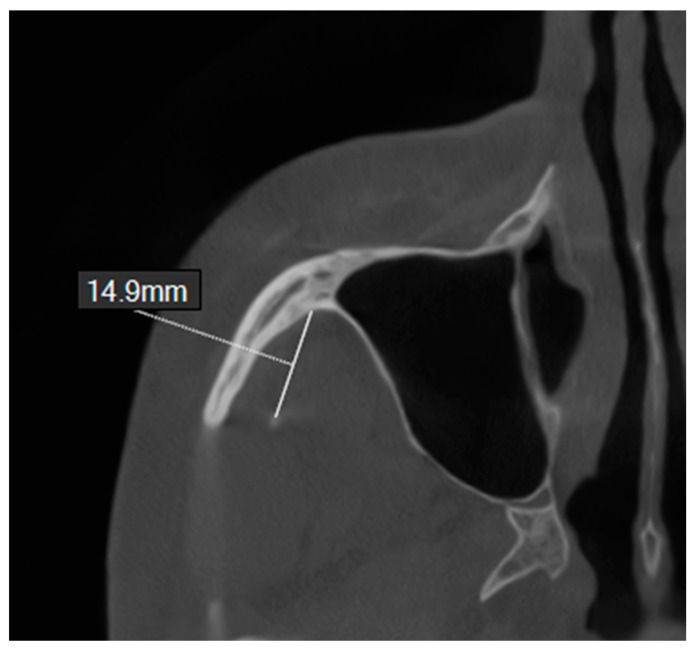
Distance between the tip of the coronoid process and the posteromedial surface of the zygomatic bone (Co-Z).

**Figure 3:**
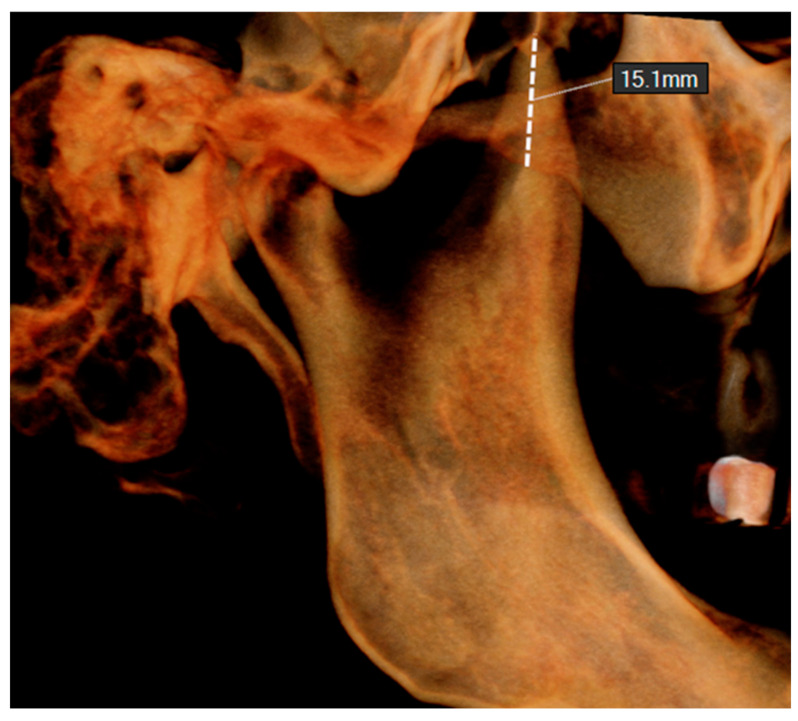
Distance of the tip of the coronoid process from the zygomatic arch (VL).

5. Coronoid/condyle ratio (Cor-L/Con-L). The ratio between the length of the coronoid and condylar process was calculated. When the ratio was >1, the coronoid process was considered hyperplastic.
6. Distance of the coronoid process from the posteromedial surface of the zygomatic bone (Cor-Z). This was assessed in the axial reformation at the level of the tip of the coronoid process. The distance between the tip of the coronoid process and the posteromedial surface of the zygomatic bone was measured (Figure 2).

7. Vertical level of the coronoid process (VL). This was evaluated on the lateral view of the volume rendered CBCT image, using the zygomatic arch as a reference. The distance from the lower border of the zygomatic arch to the tip of the coronoid process was measured (Figure 3).

Descriptive statistics were collected, and the coronoid processes were studied based on all the features. The coronoid processes with a coronoid/condyle ratio (Cor-L/Con-L) < 1 were grouped as Group A, and coronoid processes with a coronoid/condyle ratio (Cor-L/Con-L) > 1 (Group B) were grouped separately as the coronoid hyperplasia group. A comparison of the CBCT features between both the groups was performed.

## 3. Results

A total of 408 coronoid processes from 204 CBCT scans were studied. The majority number of coronoid processes displayed a triangular shape (223), followed by rounded (117), hooked (49), flattened (17), and miscellaneous (2) types. The mean Cor-L was 13.85 mm, and the mean Con-L was 16.77 mm. The mean Cor-L/Con-L ratio was 0.84.

A total of 341 coronoid processes were above the level of the zygomatic bone. Out of these, 256 coronoid processes exhibited a Cor-L/Con-L ratio < 1 (Group A), and 85 coronoid processes displayed a Cor-L/Con-L ratio > 1 (Group B). The remaining 67 coronoid processes were below the level of zygomatic bone; hence, for them it was not possible to measure the distance of the coronoid process from the posteromedial surface of the zygomatic bone (Cor-Z) and the vertical level (VL). A comparison of the distance between the coronoid process and the posteromedial surface of zygomatic bone (Cor-Z) was performed between the Group A and B subjects. There was a statistically significant difference between the two, with a *p* value < 0.001 (Table 1).

While comparing the VL of coronoid processes of Group A and B, a statistically significant difference (*p* < 0.001) was noted (Table 2).

**Figure 4:**
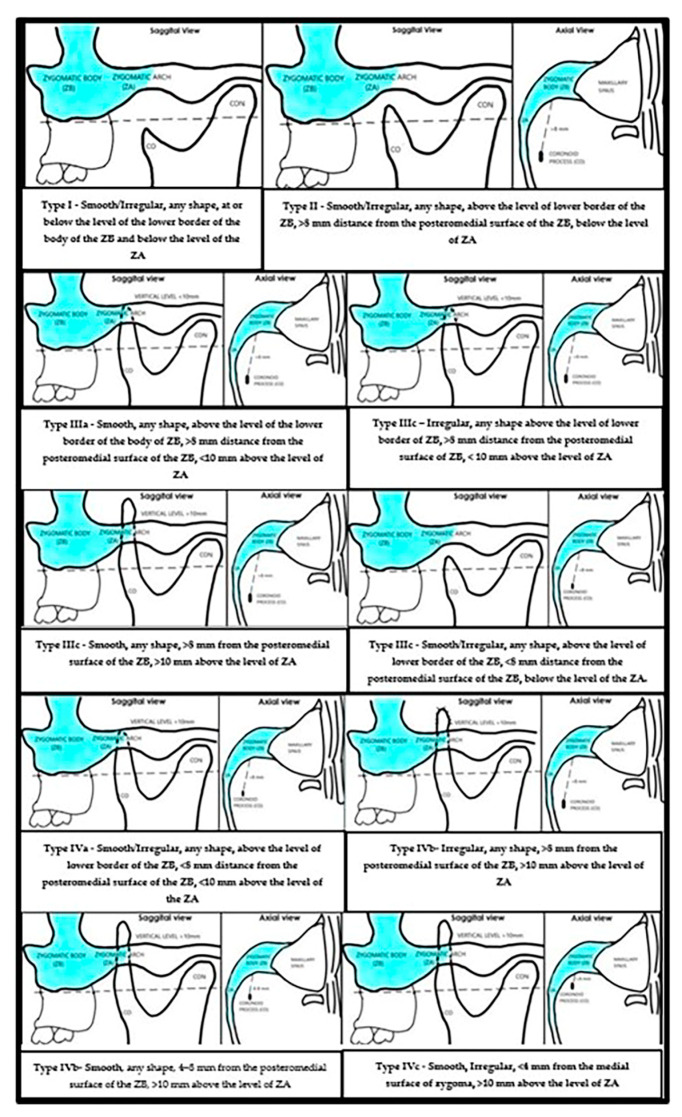
Functional classification of coronoid processes.

All the CBCT volumes were assessed by two radiologists, and the findings were recorded. The inter-rater reliability was analyzed using Krippendorff’s alpha. There was excellent correlation in the readings. Since we intend to propose a classification of the coronoid process, which has an impact on decision making in clinical situations, a consistent agreement between the observers is important to ensure reliability in the findings. A functional classification of the coronoid processes is proposed from Type I to Type IV (Table 3 and Figure 4). This classification has been proposed based on the following three criteria:

1. The surface configuration of the coronoid process, i.e., whether it is smooth or irregular.
2. The distance of the tip of the coronoid process from the posteromedial surface of the zygomatic bone.
3. The vertical level of the coronoid process. This is the distance from the lower border of the zygomatic arch to the tip of the coronoid process.

**Figure 5:**
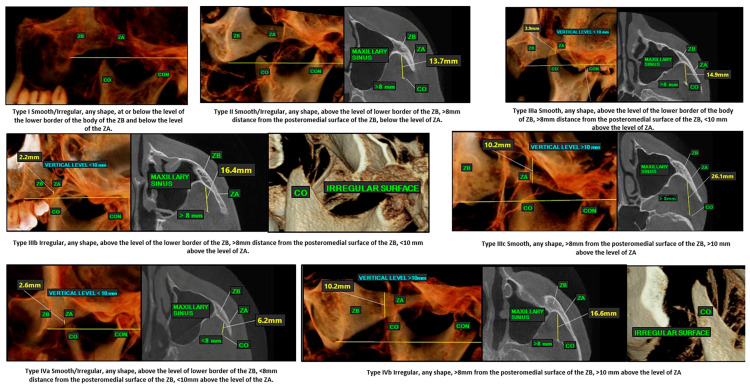
The CBCT examples of the classification types found in the study group.

Based on the proposed classification, the coronoid processes included in the current study were classified from Type I to Type IV. The most common type of coronoid process in our study group was Type IIIa = 187 (45.83%), followed by Type II = 121 (29.65%), Type I = 66 (16.17%), Type IIIc = 18 (4.41%), Type IIIb = 13 (3.18%), Type Iva = 2 (0.49%), and Type IVb = 1 (0.23%). There were no Type IVc coronoid processes in our study (Figure 5 shows the CBCT examples of the classification types found in the study group).

## 4. Discussion

In the present study, we evaluated the morphologic form (shape and surface), length of the coronoid process (Cor-L), Cor-L/Con-L ratio, vertical level (VL), and distance of the coronoid process from the posteromedial surface of the zygoma on CBCT scans in patients without any difficulty in mouth opening. The most common shape found in our study was triangular.

The current study utilizes volume rendered images and multiplanar reformatted images for assessment of the morphology of the coronoid process and its relationship to the adjacent zygomatic bone. Vanda Domingos et al. [16] concluded that 3D images were more illustrative than multiplanar reformations to diagnose and interpret coronoid process hyperplasia. Due to the isotropic nature of voxels, the CBCT enables multiplanar two-dimensional images in coronal, sagittal, oblique, and various corrected planes, without the loss of spatial resolution [17]. In this study, we leveraged this aspect to study the coronoid processes in the three orthogonal planes, along with the volumetric reconstruction, providing us with a superior vantage point in our observations. Munk, P.L., in 1989 examined the CTs of three patients with limited mouth opening; in all three patients, the coronoid process was enlarged and protruded above the inferior rim of the zygomatic arch, and in two patients, the enlarged coronoid process resulted in scalloping of the posterior surface of the zygomatic arch [18].

Coronoid process hyperplasia is considered when the length of the coronoid process exceeds that of the ipsilateral condyle and the ratio of the former to the latter is greater than or equal to 1 [11]. Stopa, Z. et al. compared the coronoid–condyle index (CCI), measured on a computed tomogram (CT), of the patients with bilateral coronoid hyperplasia with the CT-based coronoid–condyle index of the patients with no difficulty in mouth opening. The normal CCI value was up to 1.07, and a value above 1.15 was suggestive of coronoid hyperplasia [19]. In the current study, 85 out of 408 coronoid processes displayed a Cor-L/Con-L ratio > 1, yet none of these patients had any difficulty in mouth opening. This can be justified by the argument that most of the coronoid processes were at an increased distance from the posteromedial surface of the body of the zygoma, with an average distance of 15.99 mm and a range of 5.8–27.9 mm. There was a statistically significant difference between the subjects with a Cor-L/Con-L ratio > 1 (Group B) and a Cor-L/Con-L < 1 (Group A) with respect to the distance between the coronoid process and the posteromedial surface of the zygomatic bone (Cor-Z). This finding emphasizes the fact that the distance between the coronoid process and the zygoma is an important contributing factor in restricted mouth opening. One more explanation regarding the patients with elongated coronoid processes that did not display restricted mouth opening is that the coronoid process and condyle lengths were measured from a line tangent to the sigmoid notch, so the measurements will depend on the depth of the sigmoid notch and will be overestimated. Hence, we suggest that the vertical level of the coronoid process is a more reliable indicator than the length of the coronoid process.

Shujat S. et al. investigated the geometric characteristics of the coronoid process using Caucasian population-based cadaveric mandibles and revisited the definition of coronoid hyperplasia. According to them, the diagnosis of coronoid hyperplasia based on coronoid–condylar ratios could be misleading, as it mainly relies on the condylar geometry. The radiological diagnosis of coronoid hyperplasia depends on the height of the coronoid process in relation to the zygomatic arch, and the vertical growth of the coronoid process is not the only variable that is responsible for mechanical obstruction with the zygoma. When passing through the zygoma, the horizontal span is another important factor which has been disregarded [20].

We proposed a functional classification of coronoid processes considering the relationship of the coronoid process with zygomatic bone on a CBCT scan. Type IIIa was the most common (45.83%), followed by Type II (29.68%), Type I (16.17%), Type IIIc (4.41%), Type IIIb (3.18%), Type IVa (0.49%), and Type IV b (0.24%). The need for classification is clear, considering the current lack of any criteria for coronoid processes and their relationship to the adjacent zygomatic bone. Several authors suggest that restriction in mouth opening due to coronoid hyperplasia is a result of impingement of the coronoid process against the posteromedial surface of the zygomatic bone [11]. This condition is termed coronoid impingement syndrome [4]. It is desirable to have a classification criterion for the coronoid process to improve standardization when considering coronoid hyperplasia as the reason for reduced mouth opening.

Also, oral and maxillofacial radiologists should be watchful for Jacob’s disease, where the pathological elongation of the coronoid process results in the formation of a new joint with the zygomatic bone [4]. Raccampo, L. et al. conducted an extensive literature review on Jacob’s disease, reporting three cases which were successfully treated with coronoidectomy and proposed a classification of Jacob’s disease. They emphasized that multiplanar computed tomography and three-dimensional computed tomography reconstructions represent the most effective means for the correct diagnosis and surgical planning of Jacob’s disease cases [21].

The proposed classification of coronoid processes is based on the mechanical environment surrounding the coronoid process, which can have an impact on condylar motion during mouth opening. It is clinically relevant in presurgical planning, basically to determine how much of the coronoid process needs to be removed during the surgical management of an elongated coronoid process, as during a coronoidectomy. The primary objective of the detachment of the coronoid process is to relieve the interference between an elongated coronoid and the zygoma, or any articulation between them, with the effect of the contraction of the attached temporalis muscle fibers [22]. Moreover, the coronoid process is important, as it can be used as a grafting material for the reconstruction of osseous defects such as maxillomandibular fractures, alveolar defects, and sinus augmentation [20].

Warwas, F.B. et al. retrospectively reviewed data from five patients who presented hyperplasia of the coronoid process between 2018 and 2020. In all the cases, with CBCT imaging, not only could the elongation of the coronoid process be verified, but the surgery could also be planned [23].

Another advantage of advocating this classification is that while reviewing the CBCT volumes, if a dental clinician discovers that an elongated coronoid process is close to the zygomatic bone, they can be more watchful regarding the progressively reduced mouth opening in the future, since coronoid hyperplasia is an ongoing process. In addition, if the surface morphology is irregular, particularly in volume rendered images, this can raise an alarm for any likely tumors of the coronoid process, which could be discovered at an early stage and managed accordingly.

Our proposed coronoid process classification is logical and simple, grouping coronoid processes into categories that can be compared in a meaningful way. Also, this classification gives a clear indication as to when to be vigilant for diagnosing coronoid process hyperplasia as a cause for reduction in mouth opening.

The major strength of the study is that we classified the coronoid processes based on the mechanical environment surrounding them, primarily the zygomatic body and the arch. This can impact the kinematics of the coronoid process during mandibular movements. We considered three important criteria which can indicate impingement of the coronoid process against the zygomatic body: surface configuration (regular or irregular), distance of the coronoid process from the zygomatic bone, and the vertical level. To our understanding, normal bones are smooth, but if they impinge against another bone or if there is a bony pathosis, the bone becomes irregular, and this can serve as a good indicator of disease. The closer the coronoid process to the zygomatic bone, the greater the chances of impingement against the zygoma during mandibular excursions, as the space for movement of the coronoid process in the infratemporal region is reduced [1]. The length of the coronoid process above the level of the zygomatic body/arch is important, since it suggests how high the process stands and whether this dimension of the coronoid process will cause it to encounter the zygomatic body during mouth opening (Figure 5). Coronoid hyperplasia is commonly diagnosed radiologically if its height extends ≥ 1 cm above the inferior border of the zygomatic arch [20].

There are some limitations of this study, i.e., we included patients without any history of limited mouth opening and since the human subjects were not involved in the study, we did not measure the inter-incisal opening. We excluded the patients with TMJ disorders in this study, and this could impact the generalizability of the results. In their study, Isberg et al. suggested an association between internal derangement and coronoid process hyperplasia [14]. We propose an assessment of the coronoid process and its mechanical environment in patients with TMJ disorders in future studies.

The study sample is relatively small for possible generalization of the results, but it is adequate to propose a simple and practical classification. Also, age, sex, skeletal deformities, and facial form have an impact on mandibular morphology and in turn, influence the morphology of the condyle and coronoid processes. Yashmita, F.C. et al. found that the coronoid process volume was influence by sex, with women having small volumes, and the coronoid process volume was not related to either age or facial form [24]. In their study, Gomes, A. F. et al. assessed the height and volume of the mandibular coronoid process on the CBCT images of 132 patients and corelated them with age, gender, facial type, and skeletal class. They found that only gender influenced the height and volume of the coronoid process [25]. Future studies with larger sample sizes and multiple population groups must be performed to further validate or modify this classification, but it is important to bring this structure to the forefront and inform clinicians to observe this structure and study it. The impact of the mechanical environment around the coronoid process during mandibular functionality needs to be assessed. Hence, we propose a future ex vivo study to investigate the anatomic positions of the different morphologic variants of coronoid processes in relation to the zygomatic bone, from the closed mouth position to the maximal mouth opening position.

We propose one further future study recommendation. In our study sample, in one case with an elongated coronoid process, the tip was close to the petrous portion of the temporal bone; hence, if the coronoid process is situated far posteriorly, then it can impinge with the structures in the middle cranial fossa; hence, the distance of the elongated coronoid process from the structures lying posterior to it should also be evaluated.

We proposed a functional radiologic classification of coronoid processes which can help OMFRs to identify hyperplastic coronoid processes requiring attention and surgical intervention. To the best of our knowledge, no classification of coronoid processes has been reported to date. We utilized volume rendering images and axial reconstructions for assessing the morphological shape and measurements related to coronoid processes. The multiplanar reformatting of axial scans and the 3D reconstruction of CT scans permit the precise reproduction of the shape and size of the coronoid and malar structures of interest, as well as the relationships between all of the structures of the temporal and infratemporal fossae [26]. It is clear that 3D volume rendering on a CBCT can be used to evaluate bone morphology in more detail.

## 5. Conclusions

Dental professionals should include coronoid enlargement as a differential in patients with restricted mouth opening, particularly in those patients where the TMJ findings are normal. Coronoid hyperplasia is not always the sole reason for restricted mouth opening; other factors such as the vertical extension of the coronoid process beyond the lower border of the zygomatic bone/arch and the distance between the posterior surface of the body of the zygoma and the coronoid process could play a significant role in impacting mandibular movements.

## Figures and Tables

**Table 1:** Comparison of the distance between Cor-Z among Group A and B subjects (unpaired two-tailed *t*-test).

	Group A Subjects with Cor-L/Con-L Ratio < 1	Group B Subjects with Cor-L/Con-L Ratio > 1
Number of subjects	256	85
Mean (std)	15.41 (±2.919)	16.59 (±4.2) *
Std. error of mean	0.1824	0.4556

* Statistically different (*p* < 0.01), 99% confidence interval.

**Table 2:** Comparison of the VL among subjects with Cor-L/Con-L ratio > 1 and Cor-L/Con-L ratio < 1 (unpaired two-tailed *t*-test).

	Group A Subjects with Coronoid/Condyle Ratio < 1	Group B Subjects with Coronoid/Condyle Ratio > 1
Number of subjects	256	85
Mean (std)	3.606 (±2.068)	6.723 (±4.039) *
Std. error of mean	0.1730	0.4573

* Statistically different (*p* < 0.01), 99% confidence interval.

**Table 3:** Classification (based on morphology, shape, and distance from body of the zygoma and the lower border of zygomatic arch).

Type I	Smooth/Irregular, any shape, at or below the level of the lower border of the body of the ZB and below the level of the ZA.
Type II	Smooth/Irregular, any shape, above the level of lower border of the ZB, >8 mm distance from the posteromedial surface of the ZB, below the level of ZA.
Type IIIa	Smooth, any shape, above the level of the lower border of the body of ZB, >8 mm distance from the posteromedial surface of the ZB, <10 mm above the level of ZA.
Type IIIb	Irregular, any shape, above the level of the lower border of the ZB, >8 mm distance from the posteromedial surface of the ZB, <10 mm above the level of ZA.
Type IIIc	Smooth, any shape, >8 mm from the posteromedial surface of the ZB, >10 mm above the level of ZA. or Smooth/Irregular, any shape, above the level of lower border of the ZB, <8 mm distance from the posteromedial surface of the ZB, below the level of the ZA.
Type IVa	Smooth/Irregular, any shape, above the level of lower border of the ZB, <8 mm distance from the posteromedial surface of the ZB, <10 mm above the level of the ZA.
Type IVb	Smooth, any shape, 4–8 mm from the posteromedial surface of the ZB, >10 mm above the level of ZA. or Irregular, any shape, >8 mm from the posteromedial surface of the ZB, >10 mm above the level of ZA.
Type IVc	Smooth, Irregular, <4 mm from the medial surface of zygoma, >10 mm above the level of zygomatic arch.
Shapes—triangular, rounded, hooked, flattened, others. ZB—zygomatic body. ZA—zygomatic arch.	

## Data Availability

Data are unavailable due to privacy or ethical restrictions.
